# The effects of thoracic epidural analgesia on oxygenation and pulmonary shunt fraction during one-lung ventilation: an meta-analysis

**DOI:** 10.1186/s12871-015-0142-5

**Published:** 2015-11-19

**Authors:** Xiao-Qian Li, Wen-Fei Tan, Jun Wang, Bo Fang, Hong Ma

**Affiliations:** Department of Anesthesiology, First Affiliated Hospital, China Medical University, Shenyang, 110001, Liaoning China

**Keywords:** One-lung ventilation, Oxygenation, Pulmonary shunt fraction, Thoracic epidural analgesia

## Abstract

**Background:**

The aim of our study is to compare the effects of thoracic epidural analgesia combined with general anesthesia (GA) vs. general anesthesia on oxygenation and pulmonary shunt fraction during one-lung ventilation (OLV).

**Methods:**

Literature research was firstly conducted for studies related to comparison of epidural anesthesia combined with GA vs. GA with reporting of hemodynamic and oxygenation variables and published from Jan 1990 to Jan 2014 in EMBAS, MEDLINE and Cochrane Central Register of Controlled Trials databases. The studies were reviewed and data were extracted and analyzed using fixed-effect and random-effect models.

**Results:**

There are 14 trials with 60 separate comparisons enrolling 653 patients for analysis. Regarding systemic hemodynamics, thoracic epidural analgesia decreased the mean arterial pressure and mean pulmonary arterial pressure with weighted mean difference 95 % confidence interval (−6.64 [−9.57 to −3.71] vs. -6.33 [−9.25 to −3.41] and −3.18 [−5.07 to −1.28] vs. -2.05 [−3.35 to −0.75]) respectively at the two measurements time, however, only decreasing heart rate and systemic vascular resistance (−3.28 [−5.98 to −0.67] and −319.99 [−447.05 to −192.94]) over the first 30 min after OLV. For oxygenation variables, thoracic epidural analgesia is associated with significant reduction in partial arterial oxygen pressure, mixed arterial saturation of oxygenation and increased pulmonary venous admixture fraction compared to general anesthesia with weighted mean difference 95 % confidence interval (−16.52 [−21.98 to − 11.05] vs. − 14.23 [−20.81 to − 7.65]), (0.74 [0.33 to 1.15] vs. − 0.63 [−1.23 to −0.04]) and (2.53 [1.35 to 3.72] vs. 2.77 [1.81 to 3.74]) respectively before and after 30 min of one-lung ventilation. A decrease in mixed venous saturation of oxygenation occurred after 30 min of OLV (−2.39 [−3.73 to −0.99]). Besides, a higher mean value of airway pressure was found in the thoracic epidural analgesia with weighted mean difference 95 % confidence interval (1.95 [1.61 to 2.28] vs. 0.87 [0.54 to 1.20]) at the measurements.

**Conclusion:**

Based on the existing limited data puts forward recommendations for cautious usage of thoracic epidural analgesia in case of underlying risks in lower systemic hemodynamics, decreased partial arterial oxygen pressure but increases pulmonary shunt during one-lung ventilation.

**Electronic supplementary material:**

The online version of this article (doi:10.1186/s12871-015-0142-5) contains supplementary material, which is available to authorized users.

## Background

One-lung ventilation (OLV) in the lateral position is the unique character of the thoracic surgery. During the procedure, with a potential risk of increased pulmonary shunt and occurrence of hypoxemia, the physiologic defense mechanism, termed hypoxic pulmonary vasoconstriction (HPV), starts with a rapid onset [1]. HPV is generally considered to be a factor of governing the redistribution of blood flow to prevent partial arterial oxygen pressure (PaO2) from excessively decreasing and to optimize pulmonary gas exchange during OLV. This physiologic response may be altered by many factors and the effects on oxygenation or HPV should be taken into consideration when choosing the anesthetic regimen. It was suggested that the usage of thoracic epidural anesthesia (TEA) may provide adequate analgesia, reduce postoperative mortality, improve pulmonary outcomes and facilitate fast-track approach for patients undergoing thoracic surgery [2–4]. Currently, continuous TEA combined with general anesthesia has been recommended widely in patients undergoing thoracic surgery. Due to the fact that pulmonary vasculature is dominant in sympathetic tone, it may be influenced by segmental blocking the activity of the sympathetic system over the vascular pulmonary responses. However, experimental and clinical studies on controversial effects of TEA with local anesthetics or opioids on HPV response during OLV are rare [5–7]. The optimal anesthetic management during OLV has not been yet clearly determined. Therefore, our meta-analysis is aiming to address this issue based on studies published over 15 years.

## Methods

All experimental procedures listed below were approved by the Ethics Committee of China Medical University and were performed in accordance with the Helsinki Declaration for Medical Research Involving Human Subjects (the World Medical Association, Finland, created in 1964 and revised 2013).

### Literature review

This meta-analysis was performed with a prospective protocol (outline below) using recommended literature search strategies incorporating multiple search terms. The literature search was performed in EMBAS, MEDLINE and the Cochrane central register of Controlled Trials databases published from Jan 1990 to Jan 2014 for the trials related to thoracic epidural anesthesia. Thoracic epidural analgesia, thoracic epidural block or ‘epidural-general’ were combined with procedure specific search terms (one-lung ventilation, intrapulmonary shunt) and limited by Human and Clinical trials. For the TEA portion, MESH term thorax, epidural, anesthesia AND text word thoracic epidural anesthesia were used and combined with OR (*n* = 1603). MESH term thorax, analgesia, epidural and the word thoracic epidural analgesia were used and combined with OR (*n* = 1292). MESH term lung, ventilation, respiration and text word one lung ventilation were used to search the database and combined with the term OR (7902). The primary investigations of 14 trials were acquired when all search terms were combined with the term AND. Each was then further checked by another two authors for any additional studies, as were the author’s personal files for additional references that met all inclusion criteria.

### Study inclusion criteria

Thoracic epidural anesthesia (TEA) is defined as medicine delivered into the thoracic epidural space by injection or repeated bolus dosing local anesthetics (LA) or opioids. Studies given only a single epidural dose at the beginning or end of surgery (single shot) were not included.

Among all trials included in our meta-analysis, adult patients (aged > =18 years) were randomly assigned to one of the two groups: general intravenous or inhalation anesthesia (GA group); general anesthesia combined with TEA (TEA group). The observed variables are the hemodynamic changes, arterial and mixed venous blood gas analysis and the effects of on intrapulmonary shunt fraction during OLV. Non-English language reports were excluded.

Methodological qualities of included studies are graded using Cochrane scoring systems with a 5-point scale, in which a score of 1 is given for each of the following: 1) the description of the study as randomized, 2) the description of an appropriate method of randomization, 3) the description of the study as double-blinded, 4) the description of an appropriate method of double-blinding and 5) a statement of withdraws. Since nonrandomized studies are excluded, the minimum score is one and the maximum is five.

However, there are very few studies completely satisfied with all the criteria above. Hence, good-quality studies (prospective, randomized, and controlled) are included without weight by sample size. Any disputes were resolved by agreement of at least two reviewers

### Data extraction and statistical analysis

Study information was summarized and listed in Table 1. The comparisons of intraoperatively hemodynamic parameters, blood gas analysis and secondary outcomes calculated by the standard formulas, such as mixed venous blood gas analysis and pulmonary shunt fraction during OLV are included. The comparisons in trials were then analyzed independently. Patients were subgrouped by different medications in epidural catheter of TEA group or different anesthetic regimens in GA group.

**Table 1:** Included randomized controlled trials for effects of thoracic epidural analgesia on oxygenation and pulmonary shunt fraction during One-lung ventilation

Study	Participants	Interventions	Abstracted outcomes
Ozcan et al [5]	25 G-TIVA	G-TIVA/ISO: induced: fentanyl (3 μg.kg^−1^), propofol (BIS < 45) , vecuronium (0.1 mg.kg^−1^)	hemodynamic variables: HR, MAP; Intrapulmonary shunt: PvO_2_, PaO_2_,Qs/Qt
	25G-TIVA-TEA	Maintained: propofol or isoflurane respectively (according to BIS value)	
	25 G-ISO	G-TIVA/ISO-TEA: T7-T8 epidural with initial 2 % lidocaine 2 mL ,0.1 % bupivacaine +0.1 mg.kg^−1^ morphine 10 mL, then induced the same as G-TIVA/ISO group	
	25 G-ISO-TEA	Maintained: 0.1 % bupivacaine +0.1 mg.kg^−1^ morphine 7 ml.h^-1,^ and maintained with propofol or isoflurane respectively (according to BIS value)	
Garutti et al. [6]	30 G-TIVA	G-TIVA: induced: fentanyl (3 μg.kg^−1^), midazolam (2-3 mg) , propofol (2 mg/kg), rocuronium (0.6 mg/kg)	hemodynamic variables: HR, MAP; Intrapulmonary shunt: PvO_2_,PaO_2_,Qs/Qt,PaCO_2_, PH,SvO_2_,SaO_2_,CaO_2_,CvO_2;_ Other: Hb, Paw
	30 G-TIVA-TEA	Maintained: fentanyl (3 μg.kg^−1^), propfol (6–7 mg^.^kg^−1^.h^−1^); rocuronium (0.5 mg^.^kg^−1^.h^−1^)	
		G-TIVA-TEA: T6-T7 or T7-T8 epidural with initial 6–8 ml bupivacaine, then induced: same as G-TIVA group	
		Maintained: 0.375 % bupivacaine (6–7 mL.h^−1^), propofol (6–7 mg^.^kg^−1^.h^−1^), rocuronium (0.6 mg/kg)	
Jung et al. [8]	13G-TIVA	G-TIVA: induced: fentanyl (50–100 μg), propofol (4–5 μg/ml), vecuronium (0.1 mg.kg^−1^)	hemodynamic variables: HR, MAP, CVP. MPAP, PAOP, CO, SVR, PVR; Intrapulmonary shunt: PvO_2_,PaO_2_,Qs/Qt,PaCO_2_, PH, SvO_2_,SaO_2;_ Other: Hb, Paw
	13G-TIVA-TEA-B	Maintained: 20 μg/ml remifentanil, 0.2 ml.kg^−1^.h^−1^; vecuronium (2–2.5 mg^.^.h^−1^), propofol (according to BIS)	
	13G-TIVA-TEA-S	G-TIVA-TEA-B: T5-T6 or T6-T7 epidural with initial 10 ml 5 % bupivacaine, then induced: same as G-TIVA group.	
		Maintained: 0.25 % bupivacaine 0.1 ml.kg^−1^.h^−1^, propofol (6–7 mg^.^kg^−1^.h^−1^), vecuronium (2–2.5 mg^.^.h^−1^), propofol (according to BIS)	
		G-TIVA-TEA-S: T5-T6 or T6-T7 epidural with initial 10 ml 50 μg sufentanil , then induced :same as G-TIVA group	
		Maintained: 1 μg/ml sufentanil 0.1 ml.kg^−1^.h^−1^), propofol (6–7 mg^.^kg^−1^.h^−1^), vecuronium (2–2.5 mg^.^.h^−1^), propofol (according to BIS)	
Garutti et al. [9]	37 G-TIVA	G-TIVA: induced: fentanyl (3 μg.kg^−1^), midazolam (0.04 mg/kg), propofol (2 mg/kg), rocuronium (0.6 mg.kg^−1^)	hemodynamic variables: HR, MAP; Intrapulmonary shunt: PvO_2_,PaO_2_,Qs/Qt,PaCO_2_, PH,SvO_2_,SaO_2_,CaO_2_,CvO_2;_ Other: Hb, Paw
		Maintained: fentanyl (2–3 μg.kg^−1^), propfol (6–7 mg.kg^−1^.h^−1^); rocuronium (0.5 mg.kg^−1^.h^−1^)	
	35 G-TIVA-TEA	G-TIVA-TEA: T6-T7 or T7-T8 epidural with initial meperidine 2 mg.kg^−1^ diluted in avolume of 10–12 mL, then induced :same as G-TIVA group	
		Maintained: propofol (6–7 mg^.^kg^−1^.h^−1^), rocuronium(0.5 mg^.^kg^−1^.h^−1^)	
Dossow et al. [10]	25 G-TIVA	G-TIVA: induced: fentanyl (5–10 μg.kg^−1^), thiopental (3–5 mg.kg^−1^), panccuronium (0.1 mg.kg^−1^)	hemodynamic variables; HR, MAP, PAOP, MPAP, CVP; Intrapulmonary shunt: PvO_2_,SVR,PVR
	25 G-TIVA-TEA	Maintained: fentanyl (5–10 μg.kg^−1^.h^−1^), propfol (6–10 mg^.^kg^−1^.h^−1^), panccuronium(0.05–0.15 mg.kg^−1^)	
		G-TIVA-TEA: T_6–7_ or T_7–8_ epidural with initial 0.5 % bupivacaine 15 mg, then induced the same as G-TIVA group	
		Maintained: 0.5 % bupivacaine (range 15–25 mg), propfol (6–10 mg^.^kg^−1^.h^−1^), panccuronium (0.05–0.15 mg.kg^−1^)	
Feng Y et al. [11]	12G-TIVA	G-TIVA: induced :fentanyl (3 μg.kg^−1^), propfol (1 mg.kg^−^1) , Vecuronim (0.1 mg.kg^−1^)	hemodynamic variables; HR, MAP, MPAP, CVP, CO; Intrapulmonary shunt: PaO_2_, PaCO_2_, Qs/Qt
		Maintained: propfol (9–12 mg^.^kg^−1^.h^−1^), Vecuronim (0.1 mg.kg^−1^), T_7–8_ or T_8–9_ epidural with initial 1 % lidocaine 5 ml ,then maintained with normal saline 5 ml/h	
	12G-TIVA-TEA	G-TIVA-TEA: T_7–8_ or T_8–9_ epidural with initial 0.5 % ropivacaine 7–9 ml, then induced the same as G-TIVA group.	
		Maintaine with epidural ropivacaine 3–5 ml.h^−1^ combined with propfol (4.8–7.2 mg^.^kg^−1^.h^−1^), Vecuronim (0.1 mg.kg^−1^)	
Lu JH et al. [12]	10 G-SEV	G-SEV: induced: fentanyl (100 μg ), propfol (2 mg.kg^−^1), vecuronim (0.1 mg.kg^−1^)	Intrapulmonary shunt: PaO_2_, PaCO_2_, P_ET_CO_2;_ Other: Hb, Paw
	10 G-SEV-TEA	Maintained: 0.5–1.3 MAC sevofrane, panccuronium (not mentioned)	
		G-SEV-TEA: T_5–6_ or T_6–7_ epidural with initial 1.0 % lidocaine 5 ml then induced as G-SEV.	
		Maintained: epidural with 1.0 % lidocaine 5 ml.45mins^−1^, combined with sevofrane(0.5–1.3MAC), panccuronium (not mentioned)	
Wang et al. [13]	30 G-TIVA	G-TIVA: induced: fentanyl (2 μg.kg^−1^), midazolam (0.1 mg.kg-1), propfol (1.2–2.0 mg.kg^−1^) , vecuronim (0.1 mg.kg^−1^)	Intrapulmonary shunt: PaO_2_, PaCO_2_, Qs/Qt
	30G-TIVA-TEA	Maintained: fentanyl (0.2 μg.kg^−1^.h^−1^), propfol (3–6 mg^.^kg^−1^.h^−1^), vecuronium(0.05 mg.kg^−1^)	
		G-TIVA-TEA: T_6–7_ epidural with initial 1.0 % lidocaine 5 ml then induced as G- TIVA.	
		Maintained: epidural with 1.0 % lidocaine mixed with 0.25 % bupivacaine 5 ml.h^−1^, combined with propfol (3–6 mg^.^kg^−1^.h^−1^), vecuronium(0.05 mg.kg^−1^)	
Wang et al. [14]	16 G-ISO	G-ISO: induced: fentanyl (2 μg.kg^−1^), midazolam (0.1 mg.kg-1), propfol (1.0–2.0 mg.kg^−1^), vecuronim (0.16 mg.kg^−1^)	hemodynamic variables: HR, MAP; Intrapulmonary shunt: PaO_2_, PaCO_2_, PvO_2,_ Qs/Qt
	14 G-ISO-TEA	Maintained: isoflurane (2.0–4.0MAC), fentanyl (50 μg)	
		G-ISO-TEA: T_6–7_ or T_7-8_epidural with initial 2.0 % lidocaine 3 ml then induced as G- ISO.	
		Maintained: epidural with 0.5 % bupivacaine 5 ml.h^−1^, combined with propfol (4–6 mg^.^kg^−1^.h^−1^)	
Wang et al. [15]	15 G-ISO	G-ISO: induced: fentanyl (2 μg.kg^−1^), midazolam (0.05 mg.kg-1), propfol (1.5–2.0 mg.kg^−1^), vecuronim (0.1 mg.kg^−1^)	hemodynamic variables: HR, MAP; Intrapulmonary shunt: PaO_2_/Fio_2_, Qs/Qt Other: Paw
	15 G-ISO-TEA	Maintained: isoflurane (0.5–1.3MAC), vecuronim (not mentioned)	
		G-ISO-TEA: T_10–11_ epidural with initial 0.5 % lidocaine then induced as G- ISO.	
		Maintained: epidural with 0.5 % lidocaine 5 ml.h^−1^, combined with isoflurane (0.5–1.3MAC), vecuronim (not mentioned)	
Chen et al. [16]	13 G-TIVA	G-TIVA: induced: fentanyl (3 μg.kg^−1^), midazolam (0.1 mg.kg-1), propfol (1.0–2.0 mg.kg^−1^), vecuronim (0.1 mg.kg^−1^)	hemodynamic variables; HR, MAP; Intrapulmonary shunt: PaO_2_,PaCO_2_,PvO_2,_Qs/Qt, SvO_2_,SaO_2_,CaO_2,_CvO_2_, P_ET_CO_2,_ pH; Other: Paw
	13G-TIVA-TEA	Maintained: fentanyl (0.2 μg.kg^−1^.h^−1^), propfol (3–6 mg^.^kg^−1^.h^−1^), vecuronium(0.05 mg.kg^−1^)	
		G-TIVA-TEA: T_6–7_ epidural with initial 1.0 % lidocaine mixed with 0.375 % bupivacaine 8–10 ml then induced as G- TIVA.	
		Maintained: epidural with mixture of lidocaine and bupivacaine 5 ml.h^−1^, combined with propfol (3–6 mg^.^kg^−1^.h^−1^), vecuronium(0.05 mg.kg^−1^)	
Wu et al. [17]	41G-TIVA	G-TIVA: induced: midazolam (0.05 mg.kg-1), fentanil, propfol and vecuronim (no details)	hemodynamic variables; HR, MAP; Intrapulmonary shunt: PaO_2_,PaCO_2_,Qs/Qt, pH
	41G-TIVA-TEA	Maintained: fentanil, propfol and vecuronium (no details, according to BIS).	
		G-TIVA-TEA: T_5–6_ or T_6–7_ epidural with initial 0.5 % ropivacaine 7–12 ml then induced as G- TIVA.	
		Maintained: epidural with 0.5 % ropivacaine 4–5 ml.h^−1^, combined with propfol and vecuronium (no details).	
Zhang et al. [18]	43G-TIVA	G-TIVA: induced: midazolam (0.05 mg.kg-1), fentanil, propfol and vecuronim (no details)	hemodynamic variables; HR, MAP; Intrapulmonary shunt: PaO_2_,PaCO_2_,Qs/Qt,
	43G-TIVA-TEA	Maintained: fentanil, propfol and vecuronium (no details, according to BIS).	
		G-TIVA-TEA: T_5–6_ or T_6–7_ epidural with initial 0.5 % ropivacaine 7–12 ml then induced as G- TIVA.	
		Maintained: epidural with 0.5 % ropivacaine 4–6 ml.h^−1^, combined with propfol and vecuronium (no details).	
Sun et al. [19]	12 G-ISO-TEA	G-ISO-TEA and G-TIVA-TEA: induced: T_7–8_ or T_8–9_ epidural with initial 0.5 % ropivacaine 7–9 ml then induced with fentanyl (3 μg.kg^−1^), midazolam (2-3 mg), propfol (1.5 mg.kg^−1^), vecuronim (0.1 mg.kg^−1^)	hemodynamic variables; HR, MAP,MPAP Intrapulmonary shunt: PaO_2_,PaCO_2_,Qs/Qt, pH
	12 G-TIVA-TEA		
		Maintained: isoflurane and propfol respectively(no details, according to BIS).	

*TEA* thoracic epidural anesthesia with local anesthetic, opioids or both, *TIVA* total-intravenous anesthesia, *ISO* isoflurane inhalation anesthesia

Quantitative analyses were performed using Review Manager Software (version 5.0; Cochrane Collaboration, Oxford shire, England). The level of significance for all tests is set at a α level of 0.05. For dichotomous data, Petro odds ratios (ORs) with 95 % confidence intervals (CIs) were computed. When possible, data were converted to means and standard deviations (SD) for continuous outcomes and calculated as weighted mean differences with 95 % CIs between active and control groups for each study. For heterogeneity analyses: data that were not significantly homogeneous (*P* > 0.1) were analyzed with a fixed-effect model, whereas heterogeneous data (*P* ≤ 0.1) were analyzed with a random effect model. Sensitivity analyses were performed to identify sources of heterogeneity. Studies which do not report mean and SD or standard error of the mean (SEM) are not included in the meta-analysis.

## Results

### Retrieved and included studies

**Fig. 1:**
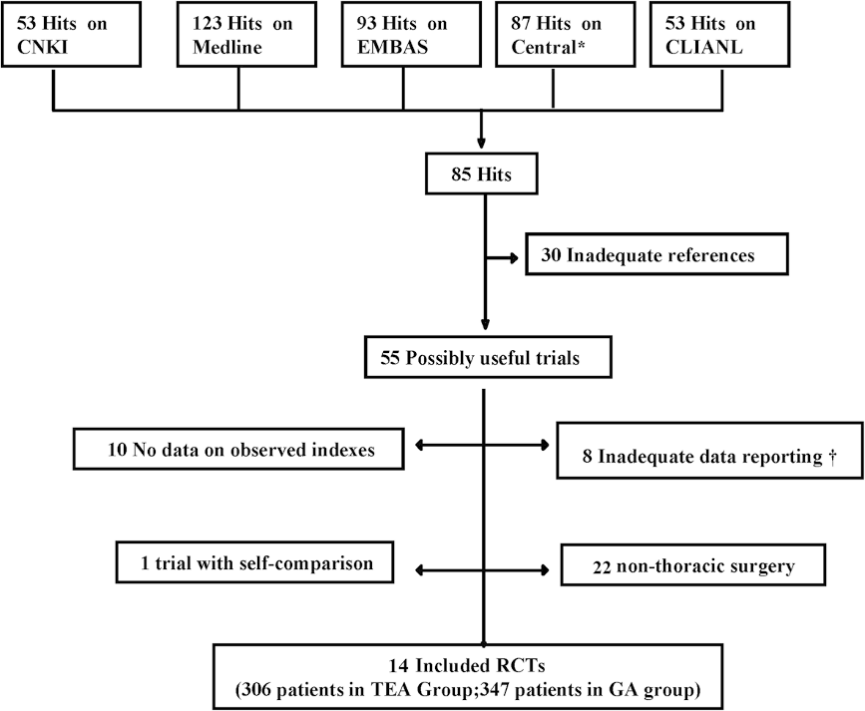
The flow diagram of retrieved, excluded, and analyzed trials. * Cochrane Central Register of Controlled Trials. †Continuous data presented as means but without standard deviation, no dichotomous data. RCT indicates randomized controlled trials

Fourteen reports were retrieved while one trial with self-comparison was rejected (Fig. 1). A total of 653 patients with 60 separate comparisons met all inclusion criteria [5, 6, 8–19] (Table 1). These reports were published between 1999 and 2010 and reported data were from 653 patients. Forty one studies were excluded: 1) not thoracic surgery; 2) no available data on observed indexes; 3) the intervention used in studies not consistent with the criteria.

Among included trials, the epidural catheter was all placed at T_6–7_, T_7–8_ or T_8–9_ interspace in the TEA group and continuously injected with local anesthetics, opioids or both via the catheter during the whole OLV procedure more than 30 min. Controls received intravenous opioids with or without nonopioid analgesics (Table 1).

### Risk of bias in included studies

Overall study quality was moderate. There was adequate randomization in 10/14 (71.4 %) of studies, double-blinding in 12/14 (85.7 %) of studies and statement of withdraw in 3/14 (21.4 %) of studies. Three studies were assessed all in three domains and five studies were assessed in two domains.

### Thoracic Epidural analgesia on systemic hemodynamics variables

Overall, it was shown that TEA did not significantly affect the changes of hemodynamic variables during two lung ventilation, and it is associated with significantly reduction in heart rate (HR) and systemic vascular resistance (SVR) only during OLV within 30 min. However, there was a continuously significant decrease in mean arterial pressure (MAP) and mean pulmonary arterial pressure (MPAP) during the whole OLV period until re-two lung ventilation (re-TLV) (Table 2).

**Table 2:** Effects of thoracic epidural analgesia on hemodynamics variables during OLV

		Median of Means (Range)			
All available data	No. of comparisons	TEA group	GA group	WMD (95 % CI)	*P* value
During two-lung ventilation					
HR(beat / min)	7	72.98 (68.80 to 83.00)	73.71 (69.00 to 78.00)	−1.33 [−5.16 to 2.50]	0.50
MAP(mmHg)	5	83.62 (76.00 to 87.00)	86.26. (75.00 to 93.00)	−2.28 [−5.51 to 0.95]	0.17
CO (L/min)	2	5.50 (5.20 to 5.80)	4.90 (4.90 to 4.90)	0.55 [−0.21 to 1.30]	0.16
CVP (cmH_2_O)	3	7.00 (6.00 to 8.00)	8.00 (6.00 to 9.00)	−1.00 [−3.94 to 1.95]	0.51
SVR (dynes · sec · cm^−5^)	3	1180.70(1022.00 to 1308.00)	1401.67(1395.00 to 1405.00)	−246.16[−408.55 to -83.77]	0.29
MPAP (mmHg)	3	20.67 (18.00 to 22.00)	18.67 (18.00 to 19.00)	2.09 [−0.94 to 5.13]	0.18
PAOP(mmHg)	2	12.50 (12.00 to 13.00)	13.00 (13.00 to 13.00)	−0.59 [−2.48 to 1.31]	0.55
During one-lung ventilation within 30 min					
HR(beat / min)	7	74.14 (66.00 to 85.00)	75.48 (73.20 to 80.00)	−3.28 [−5.89 to −0.67]	0.01
MAP(mmHg)	7	85.11 (81.00 to 89.00)	93.29 (87.60 to 100.00)	−6.64 [−9.57 to −3.71]	<0.01
CO (L/min)	2	5.40 (5.40 to 5.40)	5.70 (5.70 to 5.70)	−0.30 [−1.15 to 0.55]	0.49
CVP (cmH_2_O)	3	8.30 (7.00 to 10.00)	9.00 (8.00 to 11.00)	−0.61 [−1.72, 0.49]	0.28
SVR (dynes · sec · cm^−5^)	3	1003.33(890.00 to 1124.00)	1349.67(1141.00 to 1454.00)	−319.99[−447.05 to -192.94]	<0.01
MPAP (mmHg)	3	20.00 (19.00 to 22.00)	23.00 (23.00 to 23.00)	−3.18 [−5.07 to −1.28]	<0.01
PAOP(mmHg)	2	12.00 (12.00 to 12.00)	13.00 (13.00 to 13.00)	−1.00 [−2.87 to 0.87]	0.30
One-lung ventilation more than 30 min					
HR(beat / min)	6	76.00 (75.00 to 87.00)	75.32 (71.50 to 80.00)	−0.94 [−3.81 to 1.92]	0.52
MAP(mmHg)	6	84.12 (75.00 to 87.00)	91.85 (85.80 to 98.00)	−6.33 [−9.25 to −3.41]	<0.01
CO (L/min)	4	5.55 (4.70 to 6.10)	5.55 (5.50 to 5.60)	−0.07 [−0.64 to 0.51]	0.82
CVP (cmH_2_O)	4	7.75 (7.00 to 9.00)	8.00 (7.00 to 9.00)	−0.37 [−1.24 to 0.51]	0.41
SVR (dynes · sec · cm^−5^)	4	1168.50(981.00 to 1356.00)	1209.00(1209.00 to 1209.00)	−38.17[−201.75 to 125.42]	0.07
MPAP (mmHg)	4	20.00 (18.00 to 21.00)	22.50 (22.00 to 23.00)	−2.05 [−3.35 to −0.75]	<0.01
PAOP(mmHg)	4	12.50 (12.00 to 13.00)	13.50 (13.00 to 14.00)	−1.11 [−2.40 to 0.18]	0.09
Re-two ventilation					
HR(beat / min)	5	74.40 (65.00 to 87.00)	71.60 (67.00 to 78.00)	−0.41 [−4.08 to 3.25]	0.82
MAP(mmHg)	5	84.40 (81.00 to 87.00)	95.80 (92.00 to 95.00)	−11.83 [−15.87 to −7.79]	<0.01
CO (L/min)	2	5.55 (5.50 to 5.60)	5.20 (5.20 to 5.20)	0.35 [−0.58 to 1.29]	0.46
CVP (cmH_2_O)	3	7.67 (7.00 to 9.00)	6.33 (6.00 to 7.00)	1.20 [−0.15 to 2.55]	0.08
SVR (dynes · sec · cm^−5^)	3	1157.00(1052.00 to 1316.00)	1358.67(1226.00 to 1425.00)	−186.69[−312.37 to −61.01]	<0.01
MPAP (mmHg)	3	19.67 (18.00 to 21.00)	21.33 (21.00 to 22.00)	−1.77 [−3.61 to 0.07]	0.06
PAOP(mmHg)	2	11.00 (10.00 to 12.00)	12.00 (12.00 to 12.00)	−1.03[−2.75 to 0.68]	0.24

Data were presented as Mean and rang in bracket

### Thoracic Epidural analgesia on oxygenation and pulmonary shunt fraction variables

**Fig. 2:**
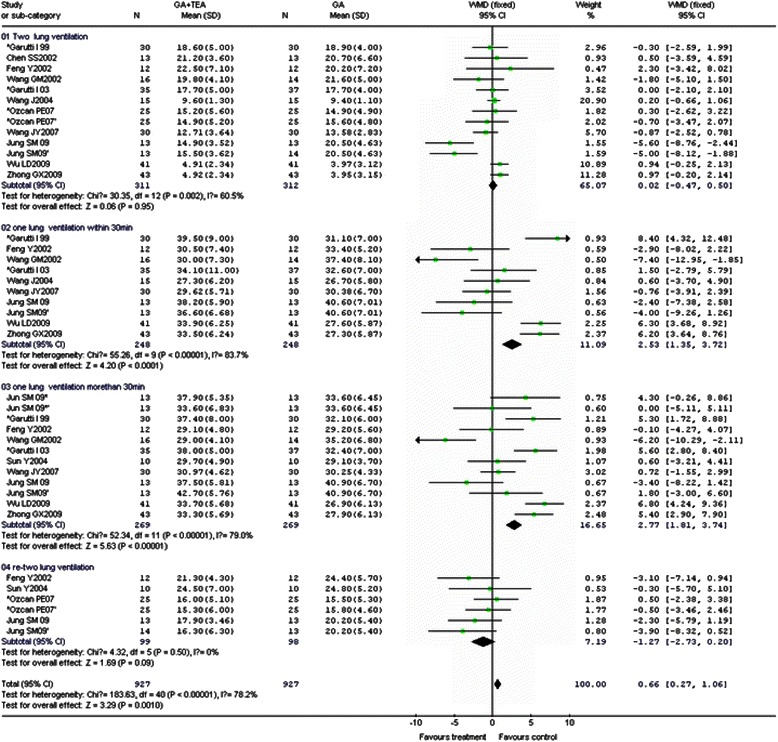
Effect of thoracic epidural analgesia (TEA) on pulmonary venous admixture fraction (Qs/Qt) in general anesthesia (GA) combined with TEA group and GA group during mechanical ventilation: Forest plot showing pooled analysis of the WMD of Qs/Qt in two groups during two-lung ventilation (01), one-lung ventilation within 30 min (02), one-lung ventilation more than 30 min (03) and re-two-lung ventilation (04) based on the fixed effects model. CI = confidence interval

It was shown that TEA did not significantly affect the pulmonary shunt fraction during TLV. From TLV to OLV, TEA modestly reduced arterial oxygen pressure (PaO_2_), mixed arterial saturation of oxygenation (SaO2) and increased the pulmonary venous admixture fraction (Qs/Qt%) and mean airway pressure (Paw) occurred during OLV. A decrease in mixed venous saturation of oxygenation (SvO_2_) occurred after 30 min of OLV (Fig. 2, Table 3).

**Table 3:** Effects of thoracic epidural analgesia on oxygenation and pulmonary shunt fraction variables during OLV

		Median of Means (Range)			
All available data	No. of comparisons	TEA group	GA group	WMD (95 % CI)	*P* value
During two-lung ventilation					
PaO_2_ (mmHg)	13	382.42(205.00 to 85.00)	377.92(191.0 to 453.00)	6.29 [0.04 to 12.17]	0.35
PaCO_2_ (mmHg)	10	39.67 (32.50 to 37.6)	40.23 (35.60 to 37.40)	−0.21 [−1.11 to 0.68]	0.64
PvO_2_ (mmHg)	7	51.50(48.00 to 55.0)	51.62(48.0 to 53.30)	0.27 [−1.51 to 2.06]	0.76
Qs/Qt (%)	13	14.80 (4.90 to 22.50)	15.50 (3.95 to 21.60)	0.02[−0.47 to 0.50]	0.95
SvO_2_ (%)	4	85.37(83.30 to 87.10)	86.68(84.90 to 88.20)	−1.22[−2.36 to −0.07]	0.58
SaO_2_ (%)	4	99.75(99.70 to 99.90)	99.45(99.00 to 99.90)	−0.06[−0.08 to −0.04]	0.30
PH	4	7.43 (7.42 to 7.43)	7.44 (7.43 to 7.44)	−0.01 [−0.02 to 0.00]	0.21
Paw (cmH_2_O)	4	18.63 (17.00 to 20.00)	19.03 (17.00 to 21.40)	−0.24 [−1.14 to 0.66]	0.60
During one-lung ventilation within 30 min					
PaO_2_ (mmHg)	10	166.90(118.00 to 211.00)	171.2(122.00 to 201.00)	−16.52[−21.98 to -11.05]	<0.01
PaCO_2_ (mmHg)	10	37.12 (36.10 to 44.20)	36.85(34.20 to 42.20)	0.29 [−0.53 to 1.10]	0.49
PvO_2_ (mmHg)	7	47.78(44.90 to 54.00)	46.14(44.50 to 51.00)	1.13 [−0.50 to 2.76]	0.17
Qs/Qt (%)	10	33.32 (27.30 to 39.50)	32.77 (26.70 to 40.60)	2.53 [1.35 to 3.72]	<0.01
SvO_2_ (%)	4	80.53(78.30 to 82.60)	81.58(78.40 to 84.20)	−1.13[−2.74 to 0.48]	0.17
SaO_2_ (%)	4	97.98(96.80 to 99.30)	97.65(97.30 to 98.00)	0.74[0.33 to 1.15]	<0.01
PH	4	7.43 (7.42 to 7.44)	7.44 (7.42 to 7.46)	−0.01 [−0.02 to 0.001]	0.41
Paw (cmH_2_O)	4	28.00 (23.00 to 32.50)	27.00 (24.00 to 30.90)	1.95 [1.61 to 2.28]	<0.01
One-lung ventilation more than 30 min					
PaO_2_ (mmHg)	11	162.27(117.00 to 203.00)	168.72(148.00 to 221.00)	−14.23[−20.81 to -7.65]	<0.01
PaCO_2_ (mmHg)	11	36.61 (33.90 to 44.60)	36.78 (35.00 to 44.20)	−0.22 [−0.96 to 0.53]	0.57
PvO_2_ (mmHg)	6	44.70(42.60 to 46.10)	44.98(43.40 to 46.60)	−0.61[−2.23 to 1.02]	0.46
Qs/Qt (%)	12	34.40 (33.60 to 42.70)	32.67 (32.10 to 40.90)	2.77 [1.81 to 3.74]	<0.01
SvO_2_ (%)	6	79.33(77.40 to 81.10)	81.97(78.50 to 83.90)	−2.39[−3.73 to −0.99]	<0.01
SaO_2_ (%)	6	97.68(96.60 to 98.10)	98.20(97.20 to 99.00)	−0.63[−1.23 to −0.04]	0.04
PH	6	7.43 (7.43 to 7.44)	7.44 (7.41 to 7.46)	0.00 [−0.01 to 0.01]	0.50
Paw (cmH_2_O)	6	26.80 (24.00 to 32.50)	26.72 (24.00 to 31.40)	0.87 [0.54 to 1.20]	<0.01
Re-two ventilation					
PaO_2_ (mmHg)	7	322.28(173.00 to 482.00)	307.28(168.00 to 407.00)	11.54[−4.25 to 27.34]	0.15
PaCO_2_ (mmHg)	3	37.8 (34.00 to 46.10)	35.6 (34.90 to 36.90)	−0.60 [−3.03 to 1.83]	0.63
PvO_2_ (mmHg)	5	47.82(43.00 to 50.00)	48.68(46.20 to 51.00)	0.06[−2.24 to 2.36]	0.96
Qs/Qt (%)	6	18.55(15.30 to 24.50)	20.15(15.50 to 24.80)	−1.27 [−2.73 to 0.20]	0.09
SvO_2_ (%)	2	81.90(81.50 to 82.30)	85.30(85.30 to 85.30)	−3.62[−6.28 to −0.95]	0.80
SaO_2_ (%)	2	99.75(99.70 to 99.80)	99.00 (99.00 to 99.00)	0.75[0.52 to 0.98]	0.67
PH	2	7.42 (7.41 to 7.42)	7.42 (7.42 to 7.42)	0.00 [−0.03 to 0.02]	0.77
Paw (cmH_2_O)	2	19.00 (18.00 to 20.00)	18.00 (18.00 to 18.00)	0.95 [−0.81 to 2.71]	0.29

Data were presented as Mean and rang in bracket

To overcome clinical heterogeneity, data were pooled for sensitivity analysis between different techniques of general anesthesia (e.g. total intravenous anesthesia vs. balanced anesthesia) and types of medications in epidural catheter of TEA group. There were no obviously differences in all observed variables. However, no pooling of data from epidural anesthesia with different local anesthetics was possible for small number of studies.

## Discussion and conclusions

The anesthetic technique is one of several factors that can affect oxygenation and hemodynamics during one-lung ventilation (OLV), among which thoracic epidural analgesia (TEA) has demonstrated to provide statistically better acute pain relief after thoracotomy and now widely used in clinic [2, 3]. However, there were few and contradictory studies considering the effects of TEA on hemodynamics and oxygenation changes during the procedure of OLV [5, 6, 9]. This is the first meta-analysis comparing the effects of TEA on oxygenation and pulmonary shunt fraction during OLV. The most significant finding of our meta-analysis is an equivalent effect of combining TEA with any technique of general anaesthesia (GA) on hemodynamic and oxygenation variables in patients undergoing OLV after re-conversion to TLV in supine position, having positive effects on patient safety during surgery. However, the current study demonstrates that TEA inhibited hypoxic pulmonary vasoconstriction (HPV) as producing larger shunt fractions and lower PaO_2_, accompanied with decreased systemic hemodynamics compared with GA after undergoing OLV for more than 30 min. It is consistent with recent studies, in which the authors showed that, an increase in Qs/Qt was accompanied with a decrease in PaO2, but cardiac output (CO) and pulmonary artery pressure (PAP) were preserved between the groups [7, 20, 21]. At the same time, TEA was found to be associated with lower MPAP in line with decreased MAP and SVR, whereas CO was comparable. Ephedrine is a partial α-and ß-agonist. All trials included in our meta-analysis showed a tendency towards lower mean arterial blood pressure in the TEA group, which was treated by administering a dose of 5 or 10 mg ephedrine intravenously. Mechanism leading to less marked effects on CO was attributed to the higher incidence of ephedrine use in TEA groups. It had significant vasopressor activity in the pulmonary vascular bed that predominantly mediated by α-adrenergic receptor activation, although ephedrine dose less than 0.15 mg/kg did not increase the intrapulmonary shunt during OLV [8, 10]. Additionally, increasing in HR and ventricular contractility strengthen by ß-adrenergic subtype activation in left ventricular tissue could also explain the similarity of the compared values of CO [10]. Hence, the decrease in oxygenation was secondary to the effect of TEA on HPV and probably not on the changes of CO.

The pulmonary vasculature is dominant in sympathetic activity by the norepinephrine released from sympathetic nerve endings [6, 9, 22]. Potential mechanism of the influence of pulmonary shunt fraction during OLV was prone to cardiovascular and hemodynamic effects of TEA. Decrease in HR, MAP, stoke volume due to blockade of sympathetic activity over the vascular pulmonary responses was shown closely associated with decreased PaO_2_ [6, 9]. The decreased PaO_2_ may further have an opposite effects on HPV [5, 6, 23]. It may produce vasodilatation of the pulmonary vessels by blocking the activity of the thoracic sympathetic response [24] or stimulate precapillary vasoconstriction via a pathway involving NO and/or cyclooxygenase synthesis inhibition [17]. In an animal study by Ishibe, TEA was demonstrated to affect the ventilation/reperfusion relationship by stimulating precapillary vasoconstriction to redistribute pulmonary blood flow away from hypoxemic lung regions to the other well oxygenated areas of the lung [7], which was consistent with the study of Garutti I [6].

However, similar but significant differences in magnitude of changes in pulmonary hemodynamic circulation accorded with systemic in both groups may contribute to the analgesic effect of TEA or the systemic effects of the absorption of the local anesthetics for overall reduced sympathetic tone, and blockade of cardiac accelerator fibers [6]. The principal weakness of our meta-analysis is that we combined data for comparison even with some studies which were heterogeneous such as epidural treatments with different drugs (e.g. local anesthetics, opioids etc.) or different patient population. On the other hand, more significant differences might attain when better designed published trails with large sample size become available.

Another main reason for failing to show the beneficial effect of TEA during OLV was that in clinical circumstances, there were several other factors affecting HPV in different direction, finally resulting in clinically significant net effects. In our study, TEA was associated with higher mean airway pressure in the dependent lung compared with GA, which may counteract HPV in the non-dependent lung by diverting blood flow away from the ventilated lung, thereby increasing the pulmonary shunt. Besides, the decreased SvO_2_ in the TEA after 30 mins of OLV in addition to changes in shunt fraction may better explain the mechanism of our obverted oxygenation changes. Furthermore, there is essentially uncharged PaCO_2_ in both groups. Though, the efficacy of HPV in hypoxic lung regions is increased in the presence of respiratory acidosis and inhibited by respiratory alkalosis. There is no net benefit to exchange gas during OLV from hypoventilation for the hypercapnia. It seems to act as a vasoconstrictor by selectively increasing ventilated lung pulmonary vascular resistance (enhanced directly regional HPV) during OLV [25]. Additional, patients having right-sided thoracotomies tend to have a larger shunt and lower PaO_2_ during OLV. This is because the right lung is larger and normally better perfused than the left [26]. The unwarranted effects of higher FiO_2_ ratios have been shown to atelectasis even after very short periods of ventilation [27, 28]. There were no observed significant differences in the other oxygenation or hemodynamic variables in our study.

Some limitations are inherent to our meta-analysis. Firstly, it is possible we have missed trial that satisfied the inclusion criteria, and some data have to be excluded as the reports are incomplete. Secondly, the quality of the randomized trials in the systemic review is varied. So few alternative protocols with small sample sizes have been studied in effects of TEA on oxygenation and pulmonary shunt fraction during OLV, and quantitative analyses were limited as a result of heterogeneity and outcomes measures. Epidural thoracic anesthesia can be performed with LA, opioids or both, which limits the studies with homogeneous design from which data can be pooled. The sensitivity analyses were only chosen according to the different anesthetic regimens of GA group, although it is unlikely that different subgroups would have changed our findings, such as re-subgrouped by the different medication of TEA group, use of right arterial blood samples instead of pulmonary arterial blood [5, 29] or different sympathetic block level. Thirdly, the time intervals for outcome assessments were chosen with principle of the greatest degree of inclusion. Although the findings are reported as statistically significant, they are very discrete in clinical terms. It might be a possibility of different results because different measurements were evaluated after OLV, but it is still difficult to draw definite conclusions until further large, well conducted trials are performed.

Nevertheless, the analyses performed with limited studies allowed us to put forward recommendations for cautious usage of TEA in counteracting HPV undergoing OLV by producing a larger shunt and a decrease in oxygenation during the OLV as the vasodilatation caused by sympathetic blockade.

## Additional file

Additional file 1: **PRISMA 2009 checklist.** (DOC 68 kb)

## Abbreviations

CIs: Confidence intervals
CO: Cardiac output
GA: General anesthesia
Hb: Hemoglobin
HR: Heart rate
HPV: Hypoxic pulmonary vasoconstriction
MAP: Mean arterial pressure
MPAP: Mean pulmonary arterial pressure
OLV: One-lung ventilation
Ors: Peto odds ratios
Paw: Mean airway pressure
PaO2: Partial arterial oxygen pressure
PAOP: Pulmonary artery occlusion pressure
Qs/Qt: Pulmonary venous admixture fraction
SD: Standard deviations
SEM: Standard error of the mean
SaO2: Mixed arterial saturation of oxygenation
SvO2: Mixed venous saturation of oxygenation
TEA: Thoracic epidural analgesia
TIVA: Total intravenous anesthesia
TLV: Two lung ventilation
WMD: Weighted mean difference
